# Nanoscale Morphology of PTB7 Based Organic Photovoltaics as a Function of Fullerene Size

**DOI:** 10.1038/srep30915

**Published:** 2016-08-08

**Authors:** John D. Roehling, Derya Baran, Joseph Sit, Thaer Kassar, Tayebeh Ameri, Tobias Unruh, Christoph J. Brabec, Adam J. Moulé

**Affiliations:** 1 Material Science Division, Lawrence Livermore National Laboratory, 7000 East Ave., Livermore, CA, USA; 2 i-MEET (Institute Materials for Electronics and Energy Technology), Friedrich-Alexander, University Erlangen-Nurnberg, Martensstrasse 7, D-91058 Erlangen, Germany; 3 Department of Chemical Engineering and Material Science, One Shields Ave., University of California, Davis, Davis,CA, USA; 4 LKS (Chair for Crystallography and Structural Physics), Friedrich-Alexander University Erlangen-Nurnberg, Staudtstrasse 3, D-91058 Erlangen, Germany

## Abstract

High efficiency polymer:fullerene photovoltaic device layers self-assemble with hierarchical features from ångströms to 100’s of nanometers. The feature size, shape, composition, orientation, and order all contribute to device efficiency and are simultaneously difficult to study due to poor contrast between carbon based materials. This study seeks to increase device efficiency and simplify morphology measurements by replacing the typical fullerene acceptor with endohedral fullerene Lu_3_N@PC_80_BEH. The metal atoms give excellent scattering contrast for electron beam and x-ray experiments. Additionally, Lu_3_N@PC_80_BEH has a lower electron affinity than standard fullerenes, which can raise the open circuit voltage of photovoltaic devices. Electron microscopy techniques are used to produce a detailed account of morphology evolution in mixtures of Lu_3_N@PC_80_BEH with the record breaking donor polymer, PTB7 and coated using solvent mixtures. We demonstrate that common solvent additives like 1,8-diiodooctane or chloronapthalene do not improve the morphology of endohedral fullerene devices as expected. The poor device performance is attributed to the lack of mutual miscibility between this particular polymer:fullerene combination and to co-crystallization of Lu_3_N@PC_80_BEH with 1,8-diiodooctane. This negative result explains why solvent additives mixtures are not necessarily a morphology cure-all.

Organic photovoltaics (OPV) have been intensely studied over the past two decades and have steadily made progress increasing the device efficiency. The breakthrough of the 10% efficiency mark has been achieved with both single and multi-junction cells^1^,^2^,^3^,^4^,^5^,^6^,^7^,^8^. Improved understanding of the local ordering and morphology of the component materials has been a large contributor to this steady improvement^9^,^10^,^11^,^12^,^13^,^14^.

A commonly studied high performing a polymer device is composed of poly({4,8-bis[(2-ethylhexyl) oxy]benzo[1,2-b:4,5-b] dithiophene-2,6-diyl}{3-fluoro-2-[(2-ethylhexyl)carbonyl] thieno[3,4-b] thiophenediyl}) (PTB7) and phenyl-C71-butric acid methyl-ester (PC_70_BM) with a processing additive diiodooctane (DIO) used for improved morphology and performance^15^,^16^,^17^,^18^. The DIO additive improves the power conversion efficiency (PCE) by at least a factor of two. The widely accepted reason for the improved performance is that the DIO decreases the component domain sizes from solution coating, thereby increasing interfacial area, exciton dissociation and the associated photocurrent^19^,^20^,^21^,^22^. DIO and other low volatility solvent additives have been widely reported in the OPV literature to decrease the polymer fullerene domain size by preventing liquid-liquid phase separation^14^. Reports on solvent mixtures always and without fail record an improvement in device function by use of a solvent additive. Due to the selection of positive results for published articles there are therefore no reports in which the use of a solvent additive results in poorer performance nor any analysis for why a solvent additive could result in a less advantageous morphology.

The nanoscale morphology of organic mixtures is very difficult to study due to lack of contrast between organic materials. Non-imaging techniques such as x-ray diffraction, neutron scattering, or various spectroscopies are typically used to infer average geometries and mixing ratios. Electron microscopy techniques are gaining popularity, but 2D images provide vertically averaged data. Tomography techniques can provide a 3D concentration map if sufficient contrast is available, if the reconstruction can avoid “missing wedge” artifacts, and if the measurement can be performed without significant beam damage. We recently used electron tomography determine the relative location and volume of three distinct phases in an P3HT:fullerene OPV device with resolution of 1.5 × 1.5 × 1.5 nm. However, in order to create sufficient contrast for the measurement, an endohedral fullerene, Lu_3_N@C_80_-PCBEH (ethyl-hexyl), was used as a replacement for PCBM (see Fig. 1 for structure)^23^. Lu_3_N@C_80_-PCBEH has a lower electron affinity (EA) than PC_70_BM which results in increased open circuit voltage (V_*OC*_) and high device efficiencies in mixtures with the donor polymer P3HT^24^. In addition, Lu_3_N@C_80_-PCBEH is an excellent contrast agent for tomography measurements allowing for detailed nanoscale morphology measurements^23^.

Unfortunately, when combined PTB7 and Lu_3_N@C_80_-PCBEH yielded very low PCE devices. In fact, Lu_3_N@C_80_-PCBEH has been reported to produce poor OPV device efficiency with all OPV polymers other than P3HT^25^. The question is why? Several reports in literature report reduced PCE in polymer mixtures with the low EA acceptor ICBA and attribute the reduced efficiency to a reduction in charge separation driving force^26^,^27^. Other reports show that mixtures of ICBA mix more intimately with PTB7, which could increase the charge recombination^28^. Alternatively, it is reported that PC_70_BM forms a more favorable morphology with PTB7 than PC_60_BM, which could be an argument that a larger fullerene structure is desired for mixtures with PTB7. This article investigates the morphological changes due to the substitution of the endohedral fullerene as well as due to different processing additives using annular dark-field scanning transmission electron microscopy (ADF-STEM) combined with electron energy loss spectroscopy (EELS), electron tomography (ET), and grazing incidence x-ray diffraction.

Since this article focuses on several electron microscopy techniques, we briefly review the origin of contrast and refer the reader to these references for more complete information. A transmission electron microscope (TEM) sends focused electrons through a thin sample and records a change in the intensity or energy of the electrons as a function of position. For a TEM the electrons are columnated over a area while for a scanning transmission electron microscopy (STEM) the electrons are focused into a <1 *Å* spot and rastered over the surface. For both techniques one can measure the bright field (total forward intensity), annular dark field (intensity of electrons scattered to high angle), or energy loss image (energy loss in a specific energy region) as a function of lateral position. The intensity in an ADF image scales approximately with the square of the atomic number so heavy atoms can be used as the contrast agent with heavier atoms appearing with higher intensity. A STEM-EELS image looks at the electron intensity at specific energy losses that correspond to specific elements. For our samples we use the energy loss for the Sulfur L-edge and for the Carbon K-edge, starting at 165 and 285 eV, respectively. EELS has a much lower signal to noise that ADF and so each pixel of each image takes more acquisition time. Supplemental Information Figure S1 shows a comparison of a STEM-EELS sulfur map, carbon map, and ADF image P3HT/Lu_3_N@C_80_-PCBEH. This film was chosen because of the small features. In a typical STEM, each electron is accelerated to 200–300 keV which upon collision with the sample causes damage by impact. This type of beam damage causes vertical shrinkage of the film due to protons being “knocked off” of the sample, referred to as “knock on” damage. The other type of beam induced damage is caused by the electrons directly breaking chemical bonds, referred to as radiolysis, this effectively causes cross-linking in polymers. Finally ET refers to the process of taking many images of the same location at different angles with respect to the beam. The images are numerically reconstructed into a 3D volume. For ET, beam damage is a serious issue because the same area is imaged over 100 times. However, due to the high signal to noise of STEM-ADF imaging, the beam current and overall dose can be drastically reduced compared to conventional TEM-ET. While our group has measured high levels of beam damage for samples imaged using STEM-EELS-ET and previous reports of TEM-ET show vertical shrinkage of 30–50%^29^, measurements of shrinkage from STEM-ADF-ET are <1% for STEM-ADF-ET imaged samples.

## Results

### Film morphology as a function of fullerene size

The standard “high efficiency” PTB7/fullerene OPV devices is spin coated from a 97:3 vol% mixture of 1,2-dichlorobenzene (DCB) and DIO^17^,^15^. The DIO preferentially solubilizes the fullerene during the film drying process and results in a smaller scale phase separation. Chloronaphthalene (CN) is also a commonly used solvent additive in OPV mixtures. Like DIO, it has a low volatility and therefore evaporates slowly from the film. However, CN solubilizes both components and is reported to allow the polymer more time to crystallize, thus leading to more pure polymer phases, the opposite effect of DIO^30^. We compare films of 1:1 by wt. PTB7:PC_60_BM, PTB7:PC_70_BM PTB7:PC_80_BEH with spin coated from DSC with the CN and DIO additives.

**Figure 2:**
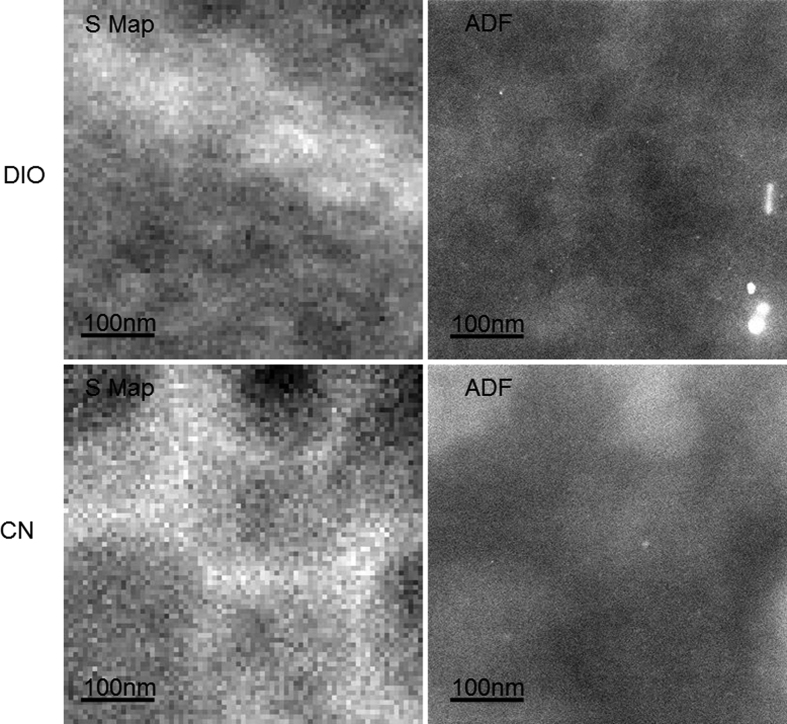
STEM/EELS sulfur maps (left) and ADF-STEM images (right) comparing PTB7:PC_70_BM films imaged over the same area. The images correspond to fabrication with 3% DIO additive (top) and 3% CN (bottom). The bright areas in the EELS images correspond to polymer-rich domains. The bright areas in the ADF images correspond to fullerene-rich domains. The fullerene clusters are smaller in the DIO additive film (∼50 nm vs. ∼20 nm).

**Figure 3:**
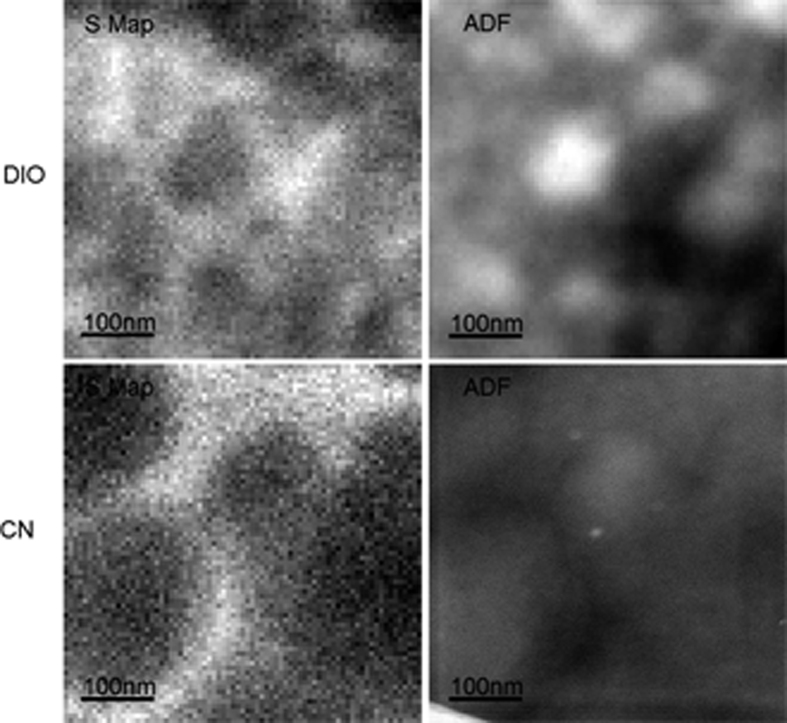
STEM/EELS sulfur maps (left) and ADF-STEM images (right) of two different PTB7:C60-PCBM films taken over the same area. The images correspond to fabrication with 3% DIO additive (top) and 3% CN (bottom). The bright areas in the EELS image correspond to polymer-rich domains. The bright regions in the ADF images correspond to fullerene-rich domains. The fullerene clusters are smaller in the DIO additive film (∼100 nm vs. ∼500 nm).

Figures 2 and 3 show STEM-EELS sulfur maps and ADF-STEM images of PTB7:PC_70_BM and PTB7:PC_60_BM films, respectively. In both figures, films processed with DIO and CN additives are compared. The sulfur maps show brighter intensity where polymer content is higher and darker regions where fullerene content is higher because only the polymer has sulfur in its structure. Conversely, the ADF-STEM images show higher intensity where fullerene content is higher and lower intensity where polymer content is higher because the fullerene has higher volumetric electron density than the polymer. These images reveal that the fullerene domains are indeed ∼50 nm when DIO is used as an additive, where the CN additive causes much larger fullerene domains. Additionally, the PC_70_BM breaks up into smaller domains than the PC_60_BM^31^. This could be another reason PC_70_BM typically performs better than PC_60_BM in devices, in addition to increased optical absorption. Our measurements confirm that there is fullerene contained in the polymer-rich regions, but, the amount was not quantified here because this has already been published^15^. Other groups have shown that the composition of fullerene in the polymer matrix is ∼30% by volume PC_70_BM in films without DIO additive (i.e. the low efficiency films)^15^,^16^,^17^.

Figures 2 and 3 demonstrate that our STEM measurements of PTB7 morphology yield domain sizes similar to that reported in the literature. These figures also demonstrate that better contrast is achieved with STEM-EELS sulfur maps than with ADF imaging in samples containing normal fullerenes.

**Figure 4:**
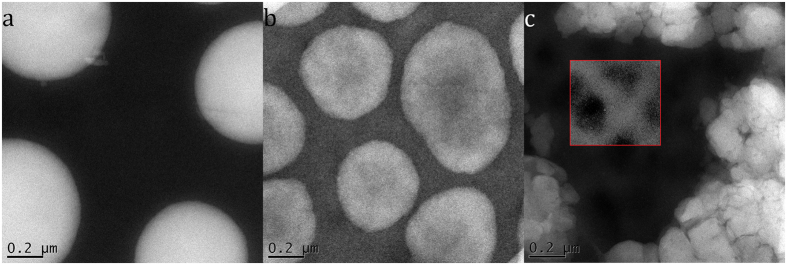
ADF-STEM images of PTB7:Lu_3_N@C_80_-PCBEH films, with no additive (**a**) 3% CN (**b**) and 3% DIO (**c**). The images were collected with the same experimental conditions, (image intensity is indicative of local fullerene content) demonstrating there is very little to no fullerene in the polymer matrix in additive free (**a**) or DIO (**c**) processed films. However, the CN additive film (**b**) contains a substantial amount of fullerene in the polymer matrix. The part of the background in the DIO image (**c**) is boxed and the image contrast is increased to show finer details.

Next, PTB7:Lu_3_N@C_80_-PCBEH films were fabricated in approximately the same *volume ratio* of polymer:fullerene as the 1:1 PTB7:PCBM weight ratio films. The density of Lu_3_N@C_80_-PCBEH has been measured to be 2.07 g/cm^3 ^32, so a 1:1.5 PTB7:Lu_3_N@C_80_-PCBEH weight ratio film has the same volume ratio as a 1:1 PTB7/PCBM film. Three films were cast with different additives; no additive, 3% by volume CN, and 3% DIO by volume. ADF-STEM images of these films are shown in Fig. 4. Due to the Lu content in the fullerenes, the contrast and practical resolution (image pixel size) in ADF imaged films, are for these samples, much higher than could be achieved using STEM-EELS.

In the additive free and DIO additive films, there are large fullerene clusters several hundred nanometers across. There is also very little intensity coming from within the polymer matrix, indicating very little to no fullerene content. Comparing the ADF attenuation coefficient^33^ in these regions to that of a pure PTB7 film shows virtually no difference, indeed indicating there is little to no fullerene in the polymer matrix. This result that Lu_3_N@C_80_-PCBEH is much less miscible with PTB7 than PC_70_BM. There are also fullerene domains (∼50–100 nm across) which are clustered together in very large aggregates (order of *μ*m) in the DIO additive film. Upon further investigation, it was determined that these fullerene aggregates are, at least, partially crystalline. This is contrary to what was found with the other PTB7:Lu_3_N@C_80_-PCBEH additive films as well as P3HT:Lu_3_N@C_80_-PCBEH where the fullerene always remained amorphous, despite extended annealing or solvent annealing^23^. The details indicates of the DIO additive morphology will be discussed in detail below.

In contrast to the no additive and DIO additive films, there is a substantial intensity in the polymer matrix in the 3% CN additive film, indicating a considerable amount of fullerene content in the polymer phase and vice-versa. Additionally, the fullerene clusters appear to have changed from domed shaped with no additive (inferred by the intensity) to blood-cell shaped with the addition of CN. The details of the CN additive morphology will be discussed in detail below.

### 3D morphology of DIO additive films

The improvements in PC_60_BM or PC_70_BM based films resulting from adding DIO raises the question of why, precisely, does DIO addition result in a decrease in material mixing in Lu_3_N@C_80_-PCBEH based films. Figure 4c show clear and substantial phase separation of PTB7 and Lu_3_N@C_80_-PCBEH. The Lu_3_N@C_80_-PCBEH domains have sharp edges suggesting the formation of crystals. The figure inset shows increased contrast in a dark region that consists of (within measurement limits) pure PTB7. The pure PTB7 film has ∼100 nm holes that correspond to thin spots. In our previous analysis of P3HT with various endohedral fullerenes, we showed that Lu_3_N@C_80_-PCBM crystallizes out of P3HT with heating and leaves behind similar thin-spots. In the previous study, we assumed that fullerene crystal formation occurred through Ostwald ripening of the crystal and simultaneous depletion of the amorphous fullerene domains. However since the diffusion rate of small molecule fullerenes is much higher than entangled polymers, the pattern of thin-spots in the polymer film indicates the pattern of depleted (removed) fullerene domains. The Lu_3_N@C_80_-PCBEH did not crystallize under any thermal conditions including long time solvent annealing with DCB and long time heating^23^. Referring again to the inset of Fig. 3c, there is a pattern of thin-spots in the polymer film with a diameter of 100–200 nm, which is consistent with the fullerene domain size found in PTB7/PC_60_BM and PTB7/PC_70_BM films that were deposited with DIO additive. This pattern suggests that Lu_3_N@C_80_-PCBEH also initially formed small domains with PTB7 upon spin coating from a mixed solvent but then later the Lu_3_N@C_80_-PCBEH was removed through an Oswald ripening process.

**Figure 5:**
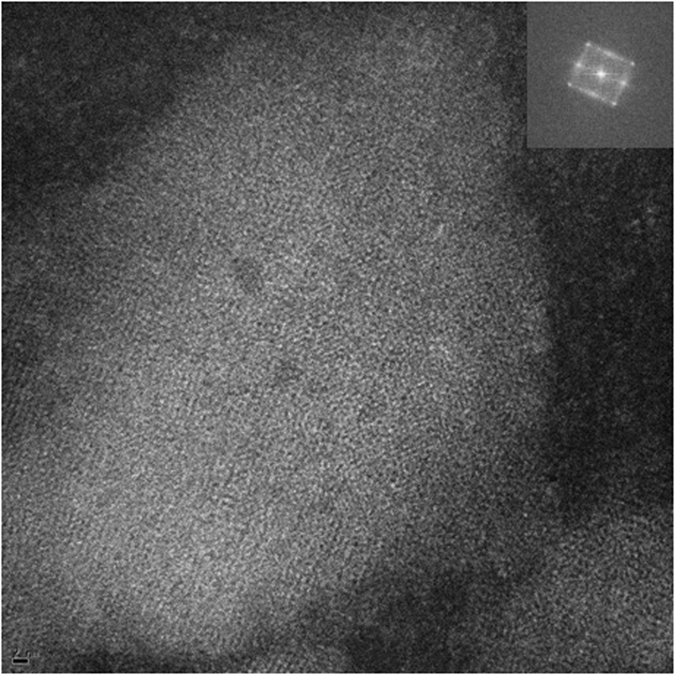
ADF-STEM image showing the co-crystal of Lu_3_N@C_80_-PCBEH:DIO with the corresponding FFT(inset).

In Fig. 5, a higher magnification image of the Lu_3_N@C_80_-PCBEH crystallites is presented. The crystalline order is clear from simple observation. The inset shows fast Fourier transform (FFT) of Fig. 5 revealing the packing nature of the fullerene crystal. The d-spacings of the 010_*f*_, 100_*f*_ and 110_*f*_ reflections are 11.05, 10.10 and 7.22 ± 0.5 Å respectively. The formation of Lu_3_N@C_80_-PCBEH crystals was very unexpected due to the previous inability of the fullerene to crystallize as a pure substance, cast and annealed with various solvents in P3HT, or cast and annealed with various solvents in PTB7^23^. In our study of Lu_3_N@C_80_-PCBEH with P3HT, the film was heated above the melting temperature and slow cooled for over 2 hours. There was not a single indication of fullerene crystallization and it was concluded that Lu_3_N@C_80_-PCBEH is less likely to crystallize that either PC_60_BM or PC_70_BM. We do not entirely rule out the possibility of pure Lu_3_N@C_80_-PCBEH crystals, but from our investigations this seems unlikely. Therefore, we speculatively conclude that the crystals observed in the DIO additive films are a co-crystal of Lu_3_N@C_80_-PCBEH and DIO.

**Figure 6:**
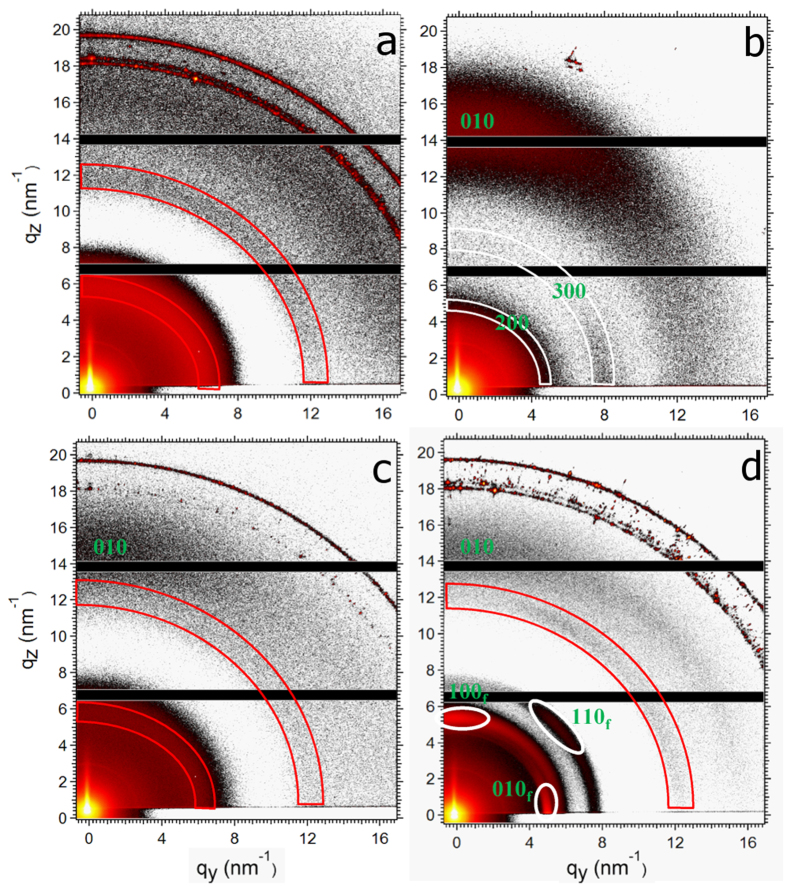
GIXD detector images of pure Lu_3_N@C_80_-PCBEH (**a**) pure PTB7 (**b**) PTB7:Lu_3_N@C_80_-PCBEH with no additive (**c**) and PTB7:Lu_3_N@C_80_-PCBEH with 3% DIO added. The red sectors refer to the scattering rings of the amorphous fullerene and the white sectors refer to the reflections of the polymer crystals. Ovals mark the diffraction spots of the proposed oriented Lu_3_N@C_80_-PCBEH:DIO crystallites.

To further investigate the structure of the DIO additive films, grazing incidence x-ray diffraction (GIXD) was performed. Figure 6 displays the diffraction patterns obtained from the pure components and mixed films. The reflections due to the PTB7 diffraction are in agreement with the previously reported pattern^34^. PTB7 lamella exhibit a preferred face-on orientation with respect to the substrate surface since the 100 Bragg-reflection is observed at 0.31 Å in the q_*y*_ direction, and the broad 010 *π* - *π* stacking peak is oriented at 1.7 Å along the q_*z*_ direction. The pattern of amorphous Lu_3_N@C_80_-PCBEH exhibits three isotropic rings originating from the form factor of the fullerene molecules. The weak isotropic scattering signal of the polymer crystals is more pronounced in neat PTB7 films than in PTB7:Lu_3_N@C_80_-PCBEH films. On a first view, the corresponding intensities in the GIXD data of the sample with DIO additive seem to be enhanced dramatically. However, a closer view reveals that the azimuthal intensity distribution of the higher order peaks 200 and 300 are inhomogeneous. Furthermore, it turns out that the inhomogeneous intensity distribution is significantly different for the 200 and the 300 reflections. The scattering intensity maximum of the 300 peak are observed at 45° whereas the maximum of the 200 peak is observed at 0°. This finding indicates that the intensities found on the 200 and 300 Debye-Scherrer-rings do not correspond to an enhanced polymer crystallinity (see supporting information for more details).

A homogeneous distribution of scattering intensity over the Debye-Scherrer-cone indicates the presence of amorphous fullerene (outlined in red) in the pure Lu_3_N@C_80_-PCBEH film, the PTB7:Lu_3_N@C_80_-PCBEH without additive film and the PTB7:Lu_3_N@C_80_-PCBEH with DIO film (Fig. 6a,c,d, respectively). However, additional Bragg-intensities of Lu_3_N@C_80_-PCBEH:DIO crystallites are observed additionally in the DIO additive film (outlined in Fig. 6d).

To resolve the origin of the extra intensity in the range of the 200 and 300 reflection, the STEM diffraction pattern for crystalline fullerene aggregates (Fig. 5) is taken into account. Assuming the formation of such fullerene crystals with a preferred orientation with the (100) plane in parallel to the substrate surface the enhanced intensities of the GIXD measurements can be explained to originate from the 010, 100, and 110 reflections of the fullerene crystals, respectively. A quantitative analysis gives d-spacings of 11.22, 9.97, and 7.57 Å for the 010_*f*_, 100_*f*_, and 110_*f*_ reflections, respectively, which is in good agreement to the values found by TEM analysis (cf. above). In a previous study of an endohedral fullerene with the same cage Lu_3_N@C_80_·(o-xylene) the lattice parameters a and b were determined to be 10.99(19) Å and 11.09(19) Å, respectively, which also nicely agrees to our results^35^. The fullerene crystals are obviously well ordered with respect to the substrate, which suggests that the fullerene crystallites are oriented to the face-on orientation of the PTB7 crystallites.

### 3-D morphology of CN additive films

**Figure 7:**
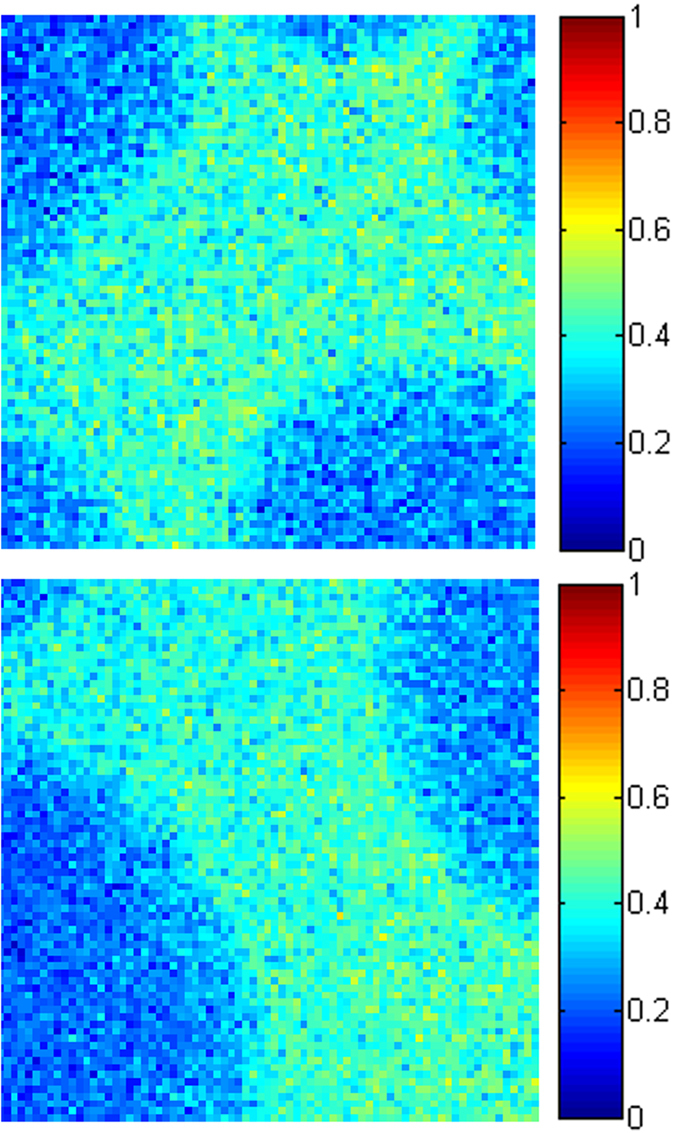
Concentration maps of PTB7 in the CN additive films. The two maps have an average concentration of PTB7 in the PTB7-rich areas of 59 vol%. The PTB7 content in the fullerene-rich domains is ∼22 vol%.

The PTB7:Lu_3_N@C_80_-PCBEH films cast with no additive and with DIO additive (see Fig. 4) have almost no fullerene in the polymer matrix while the CN additive films show some mixing and also flattened fullerene rich domains. The darker center of the fullerene rich domains could indicate either reduced fullerene content or a reduction in layer thickness at the center of the fullerene domains. To determine if the fullerene domains have changed shape (rather than concentration) the sample thickness and ratio of polymer to fullerene must be determined at each location. A quantitative measurement of vertical fullerene concentration was made by comparing the sulfur signal from the mixed film to that of a pure PTB7 film and then dividing by the film thickness determined from the low loss plasmon (see Supporting information for more details). Kesava *et al.* previously measured the polymer:fullerene content of a mixed organic film successfully using EELS, but the normalization used here differs from that study slightly due to different film conditions^36^. Figure 7 shows the composition of the CN additive film, the colors represent the fraction of PTB7 present; two separate images of different locations are presented to show that the measurement is consistent. Large fullerene clusters (dark blue in Fig. 7) are present in both images, just as in seen in the ADF images of Fig. 4b. The PTB7 rich areas contain an average volume composition of 60 vol% PTB7: 40 vol% fullerene, where the fullerene-rich areas contain approximately 80% fullerene: 20% PTB7. This result is close to what is expected for the miscibility of PC_60_BM in PTB7 without a DIO additive^15^,^16^,^17^. These images revealed that the fullerene domains are thinner at the center and uniform in concentration. Fine details of the morphology in either PTB7 and Lu_3_N@C_80_-PCBEH domains cannot be resolved due to vertical averaging of the signal.

The CN additive film has an average of roughly 40% Lu_3_N@C_80_-PCBEH in the PTB7 matrix which should make a decent PV cell. However, the 2D images tell very little about the local morphology of fullerene in this region. To determine how the fullerene is distributed three-dimensionally, electron tomography utilizing the discrete algebraic reconstruction technique (DART) was used, as in previous work (see supporting information for more details)^23^,^32^. DART has been demonstrated to reduce some reconstruction artifacts, especially those related to the missing wedge of information due to missing tilt angles and to aid in the assignment of relative gray levels in mixed samples^37^,^38^.

**Figure 8:**
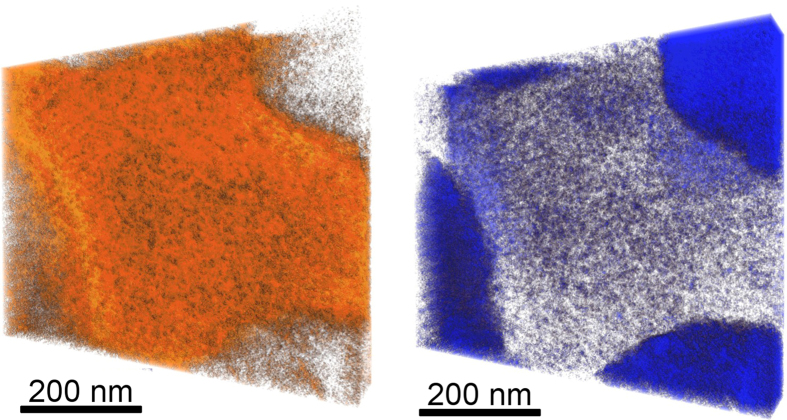
Tomographic reconstruction of PTB7(left) and Lu_3_N@C_80_-PCBEH (right) from ADF-STEM tilt-series. The reconstruction reveals the presence of small islands of fullerene which are connected, but not well connected to the electrodes. The presence of these small domains is likely the reason for the higher J_*SC*_ of the CN additive films, but also the reason for their poor overall performance (compared to PTB7:PCBM), as the domains are not completely continuous to the electrodes.

Figure 8 shows the reconstruction of the two phases of a CN additive film, PTB7-rich (left) and fullerene-rich (right). The reconstruction reveals that the fullerene in PTB7-rich domains is not dispersed molecularly, as expected for PTB7:PC_70_BM, but is aggregated in very small (<5 nm) clusters. This clustering is consistent with low miscibility of Lu_3_N@C_80_-PCBEH in PTB7 seen from the additive-free film. The clusters of fullerene are only partially connected but may or may not be percolating (this is beyond the resolution capability of the ET reconstruction). The lack of a percolation network to the cathode would lead to poor charge collection in this film. The PTB7 phases in the fullerene rich domains also appear granular which means that the PTB7 is randomly dispersed in the fullerene and does not form any kind of internal structure.

The tomographic reconstruction is centered between four Lu_3_N@C_80_-PCBEH domains and represents the most co-planar location on the sample that we could locate. A DART reconstruction of a slab geometry film requires the assumption of complete co-planarity of the film interfaces and deviations from this are seen as reconstruction artifacts^32^. Evidence of these artifacts can be seen as blue shadows on the right and left side of the PTB7 rich domain near the Lu_3_N@C_80_-PCBEH domain edge. This shadow comes from changes in film thickness that cannot be numerically accounted for. Also, unlike in our previous work^23^,^39^, the phase composition cannot be accurately calculated because the average composition of the imaged area is unlikely to be the average composition of the film, due to the large fullerene aggregates.

### Correlation with Device Characteristics

Table 1 shows the device characteristics of PTB7:Lu_3_N@C_80_-PCBEH devices fabricated without additive and with 3% DIO and 3% CN additives. Surprisingly, the film with no additive performs the best overall. Even this device only shows a PCE of 0.4%, which is more that 20× lower than a high efficiency PTB7 OPV device. The DIO additive device is by far the worst as could be expected by the complete and large scale phase separation caused by the crystallization described above. For both the no-additive and 3% CN additive devices the Lu_3_N@C_80_-PCBEH fullerene increases the V_*OC*_ by 0.15–0.3 V compared to devices fabricated using PC_60_BM or PC_70_BM. The increased mixing caused by addition of the CN additive does cause a relative increase of the J_*SC*_ with respect to the no-additive sample, but not nearly as much as would be expected given the change in concentration.

The low J_*SC*_ which is generated in PTB7:Lu_3_N@C_80_-PCBEH devices is certainly largely affected by the morphology. Evidence of reduced generation of separated charges at the polymer:fullerene interface using an endohedral fullerene compared to C_60_ or C_70_ has also been reported. Liedtke *et al.* demonstrated reduced charge generation by endohedral fullerenes by comparing the photoluminescence (PL) quenching of a P3HT:Lu_3_N@C_80_-PCBEH mixture to a P3HT:PC_60_BM mixture, showing that the quenching was lower in the endohedral fullerene mixture^40^. To investigate this possibility, the PL was measured for films of pure PTB7, PTB7:PC_60_BM, PTB7:Lu_3_N@C_80_-PCBEH with no additive, PTB7:Lu_3_N@C_80_-PCBEH with 3% CN added and PTB7:Lu_3_N@C_80_-PCBEH with 3% DIO added. The PL data is shown Supplemental Figure S7.

## Discussion

The data presented above investigates the use of Lu_3_N@C_80_-PCBEH as an electron acceptor for OPV devices using the polymer PTB7 as a donor. Lu_3_N@C_80_-PCBEH is particularly interesting because (1) its reduced EA compared to other fullerenes, which could increase the V_*OC*_ of OPV devices and (2) the metal center gives scattering contrast for electron microscopy that would allow nanoscale morphology measurements and (3) PTB7 showed better mixing with PC_70_BM than PC_60_BM which suggests that larger fullerenes could be an advantage.

Like many high performance co-polymers, the performance of OPV device mixtures with PTB7 and fullerenes is greatly improved by using a solvent additive in the casting mixture and in particular DIO. We show here that Lu_3_N@C_80_-PCBEH forms a co-crystal with DIO that causes the Lu_3_N@C_80_-PCBEH to be leeched out of the polymer film and thereby destroys the layer morphology. The inset to Fig. 3c provides insight into the mechanism for the crystal formation. The polymer film is shown to have thin spots that most likely were formed during the film formation process by the presence of Lu_3_N@C_80_-PCBEH rich domains. The domain size is typical of PC_70_BM fullerene films solution cast with a DIO additive. In other words the DIO additive seems to initially work as an emulsifier for Lu_3_N@C_80_-PCBEH into PTB7. DIO has a much lower vapor pressure than the main solvent, DCB, and so as the film dries, the DIO content is enriched. Typically (and also here) the film is placed onto a hot plate at ∼50 °C to drive off the solvent more quickly. At this time it appears that crystals of Lu_3_N@C_80_-PCBEH nucleate. The orientation of the crystals is the same as the PTB7 polymer (Fig. 6) and so it is likely (though not proven) that these co-crystals nucleate on the PTB7 polymer. The co-crystals of Lu_3_N@C_80_-PCBEH and DIO are enlarged by transport of the Lu_3_N@C_80_-PCBEH through film that is swollen with DIO. The PTB7 is depleted of fullerene leaving behind thin spots where the Lu_3_N@C_80_-PCBEH clusters initially formed. Since the PTB7 is only solvated by the DCB solvent and its T_*g*_ is greater than 50 °C, it does not diffuse. This formation process of Lu_3_N@C_80_-PCBEH/DIO co-crystals explains why Lu_3_N@C_80_-PCBEH does not make good devices with most co-polymers that use DIO as a solvent additive. This is also a clear example that shows that DIO is not always an advantageous addition as it can lead to the formation of co-crystals.

We also examined the use of CN as a solvent additive. This solvent additive increased the miscibility of Lu_3_N@C_80_-PCBEH with PTB7 but phase separation on the micrometer length scale is still evident. As previously reported, the PTB7 polymer does not form pure polymer domains, but instead forms a mixed polymer/fullerene phase^15^. Figure 7 shows that PTB7 rich-domains are ∼40% fullerene by volume, which is theoretically enough to assure percolation of electrons to the electrodes. Figure 8 shows that the Lu_3_N@C_80_-PCBEH forms clusters within the PTB7 that may not be connected. The CN additive films had significantly lower fluorescence quenching than films without additive, but the improvements in J_*SC*_ were only moderate and the V_*OC*_ is reduced. These results suggest that recombination between PTB7 and Lu_3_N@C_80_-PCBEH is high and dominates the IV characteristics. One reason for high recombination could be that electrons are trapped in Lu_3_N@C_80_-PCBEH domains that do not connect to electrodes. Another could be poor charge separation due to insufficient electronic driving force. Finally, the Lu_3_N@C_80_-PCBEH rich domains dominate over 50% of the surface and have very little (∼20%) PTB7 content, so at least half of the film is not an effective photovoltaic device.

## Conclusions

The morphology of mixed layers of the high-performance polymer PTB7 and an endohedral fullerene, Lu_3_N@C_80_-PCBEH, were investigated. The device performance was found to be inferior to PTB7:PCBM (either C_60_ or C_70_), even when using the widely accepted performance enhancing additive, diiodooctane. Electron microscopy studies revealed that in films with diiodooctane added, the fullerene strongly aggregated into micrometer sized crystals. Further investigation shows that the DIO initially solvates the Lu_3_N@C_80_-PCBEH but as the DIO slowly evaporates, co-crystals of Lu_3_N@C_80_-PCBEH and DIO form and ripen. The solvent additive chloronaphthalene causes the Lu_3_N@C_80_-PCBEH to partially disperse in the polymer matrix, but only in small, poorly connected clusters. The poor connectivity reduces the charge collection ability of the mixture compared to PC_70_ and PC_60_BM. Additionally, Lu_3_N@C_80_-PCBEH is less miscible with PTB7 than smaller fullerenes, leading to less effective quenching of PTB7 fluorescence than with a similar volume of PC_60_BM.

This work demonstrates important aspects of component mixing in organic photovoltaic mixtures. In particular, it is necessary that the fullerene component not crystallize because crystallization coupled with low vapor pressure solvents leads to phase separation of polymer and fullerene. Solvent additives and DIO in particular are universally credited with improved BHJ morphology. Here is an example in which the solvent additive causes phase separation through co-crystallization rather than allowing increased miscibility.

## Experimental

### Device Fabrication

Bulk-heterojunction devices were fabricated using PTB7 as the electron donor and Lu_3_N@C_80_PCBEH as the electron acceptor. Poly(3,4-ethylenedioxythiophene) polystyrene sulfonate(PEDOT:PSS) was used as a hole transport layer. The standard device structure was as follows: ITO/PEDOT:PSS (AL4083)/PTB7: Lu_3_N@C_80_-PCBEH/Ca/Ag. ITO coated glass with a sheet resistance of 12.3 Ω/sq (Osram) was used as the transparent electrode. ITO was cleaned by ultrasonic treatment with acetone and isopropyl alcohol and dried under flow of dry nitrogen. A PEDOT:PSS (AL4083,H.C. Stark) solution was bladed at 50 °C onto clean substrates resulting in a thickness of approximately 40 nm as determined with Dektak profilometer. PEDOT:PSS layer was annealed for 15 min at 140 °C in a nitrogen filled glove box. The active layers consisting of PTB7 (7 mg/ml) and Lu_3_N@C_80_-PCBEH (12 mg/ml) were stirred at 80 °C for 12 h before use. The active layers were spin coated from o-DCB solution with different weight ratios onto the PEDOT: PSS layer. The samples were transferred to glove box and left under nitrogen atmosphere for overnight. A Ca (∼15 nm) and Ag (∼85 nm) top electrode was evaporated via a mask in vacuum onto the active layers with an electrode area of 0.104 cm^2^. PCE was calculated from J-V characteristics recorded with a Botest source measure unit. Illumination was provided by an Oriel Sol1A 94061 solar simulator with an intensity of 100 mW/cm^2^ where the light intensity was calibrated with a standard silicon photodiode.

### STEM and ET

TEM specimens were prepared by floating the films off of PEDOT:PSS coated substrates in water and picking up the film with a lacey carbon coated Cu TEM grid. The films were imaged in a JEOL 2100-F at 200 kV. Tilt angles of ±70° were used for the ET study with a 1.5° saxton tilt scheme. The images were aligned manually in IMOD^41^. The reconstruction was performed using the DART algorithm within the ASTRA toolbox^42^ using 10 iterations; SIRT was used with as the internal algorithm using 30 iterations per DART iteration. The final reconstruction was determined by minimizing the projection error using two gray levels.

### GIXD

The samples were measured with the highly customized Versatile Advanced X-ray Scattering instrument Erlangen (VAXSTER) at the chair for Crystallography and Structural Physics (Universität Erlangen-Nürnberg, Germany)^43^. The system was equipped with a Cu K_*α*_ (*λ* = 1.5418 *Å*;) Microfocus X-ray source (GeniX, 30 W, Xenocs, Sassenage, France). The beam was collimated by two automated double slit systems (aperture sizes 0.7 × 0.7 mm^2^ and 0.4 × 0.4 mm^2^) with a distance of about 1.2 m. The second slit system consists of four scatterless silicon single crystal blades. The sample was positioned inside the fully evacuated beam path in front of the 2D Pilatus3 300 K detector (Dectris Ltd., Baden, Switzerland). All GIXD measurements were performed at 22.2 °C. The collimation line was tilted and shifted with respect to the horizontal plane allowing grazing incidence angles which maximizes the scattering volume and enhances the scattered intensity. The incident angle *α* was set to 0.21 ± 0.032° which is just below the critical angle of total reflection of the substrate. The sampledetector distance was calibrated to 178.5 mm using a silver behenate standard, providing a beam size of about 0.5 × 15 mm^2^ at the sample position.

## Additional Information

**How to cite this article**: Roehling, J. D. *et al.* Nanoscale morphology of PTB7 based organic photovoltaics as a function of fullerene size. *Sci. Rep.* **6**, 30915; doi: 10.1038/srep30915 (2016).

## Supplementary Material

Supplementary Information

## Figures and Tables

**Figure 1:**
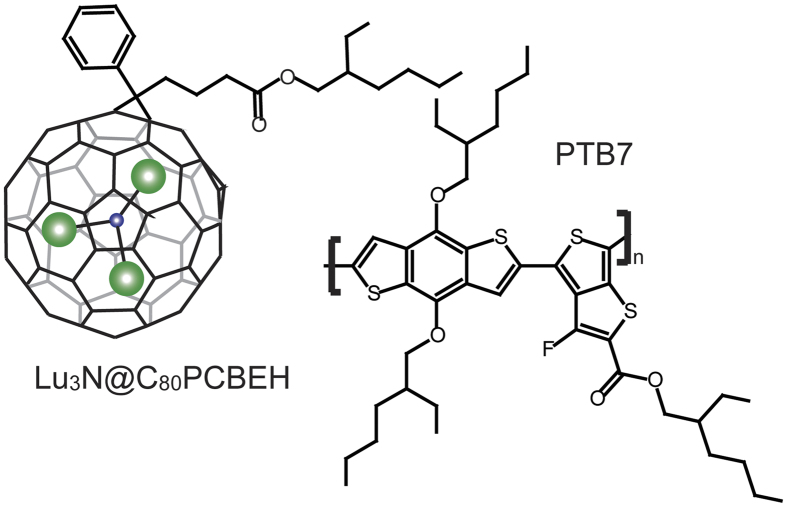
Molecular structures of PTB7 and Lu_3_N@C_80_-PCBEH.

**Table 1:** PTB7:Lu_3_N@C_80_-PCBEH device characteristics.

	w/o additive	3% DI	3% CN
J_*SC*_ [mA/cm^2^]	1.1	0.4	1.6
V_*OC*_ [V]	0.9	0.1	0.75
FF	0.42	0.26	0.43
PCE [%]	0.4	0.02	0.35
