# Supplementary material for: Nanoscale Morphology of PTB7 Based Organic Photovoltaics as a Function of Fullerene Size
Source: Sci Rep. 2016 Aug 8;6:30915. doi: 10.1038/srep30915 (PMC4976328; doi:10.1038/srep30915)
Supplement: Supplementary Information [file srep30915-s1.pdf]

Supporting information for: Nanoscale Morphology of PTB7 Based Organic Photovoltaics as a Function of Fullerene Size

John D. Roehling,† Derya Baran,‡ Joseph Sit,¶ Thaer Kassar,§ Tayebeh Ameri,‡ Tobias Unruh,§ Christoph Brabec,‡ and Adam J. Moulé*,¶

†*Material Science Division, Lawrence Livermore National Laboratory, 7000 East Ave., Livermore, CA, U.S.A.*

‡*i-MEET (Institute Materials for Electronics and Energy Technology), Friedrich-Alexander, University Erlangen-Nurnberg, Martensstrasse 7, D-91058 Erlangen, Germany*

¶*Department of Chemical Engineering and Material Science, One Shields Ave., University of California, Davis, Davis,CA, U.S.A.*

§*LKS (Chair for Crystallography and Structural Physics), Friedrich-Alexander University Erlangen-Nurnberg, Staudtstrasse 3, D-91058 Erlangen, Germany*

E-mail: amoule@ucdavis.edu

Microscopy Contrast

STEM:EELS

Electron energy loss spectroscopy (EELS) studies were performed on the films in order to determine the concentration of $Lu_3N@C_{80}$-PCBEH or PTB7 within a certain area. In order to do this, the sulfur L-edge from the PTB7 was used as it was the only accessible elemental

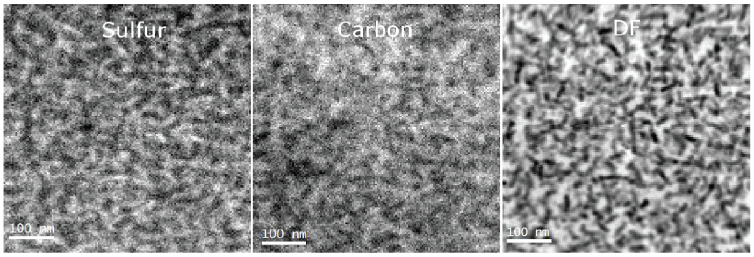

Figure 1: STEM/EELS sulfur and carbon maps from a P3HT:$Lu_3N@C_{80}$-PCBEH film along with a ADF image. All of these images were obtained simultaneously. Clearly the Dark field image gives the largest contrast between polymer (dark) and fullerene (light) domains. The next best contrast is the Sulfur map with polymer (light) and fullerene (dark) domains. Finally the Carbon map with polymer (dark) and fullerene (light) contrast gives the poorest contrast. The sulfur map is better than the carbon map because the density difference is greater (100% of the sulfur is in the polymer). For the carbon map both polymer and fullerene have high carbon content and so the density difference is small.

edge in the possible energy range that was only present in one of the components (lutetium edges are too high in energy). The signal was assumed to be linear with the thickness of PTB7 that the beam passed through. In order to get a concentration, the sulfur signal coming from a particular thickness was calibrated using a pure PTB7 film. An area of 500 nm $\times$ 500 nm was scanned for the sulfur edge and in the zero-loss region to get the thickness (number of inelastic mean-free path-lengths). The average value of the sulfur signal divided by the thickness was used as the point for 100% PTB7. The mixed film was then measured and a similar procedure applied, only this time, the thickness normalization had to be modified in order to account for the different inelastic mean-free path-lengths of the two component materials. A simple model was used by using an averaged mean-free path-length of both materials in the nominal mixing ratio of the film being investigated. Using thickness normalized sulfur edge intensity from the mixed film and dividing by the thickness normalized sulfur edge intensity of pure PTB7, maps of the polymer concentration were determined (shown in Figure 3 of the main text).

Electron tomography

As mentioned in the main text, the reconstruction algorithm used was the discrete algebraic reconstruction technique (DART). This method uses knowledge of the reconstructed sample to constrain the reconstruction to a set number of particular grey levels. This is a more accurate picture for an object with constant density/constant composition components than typical reconstructions done using only the simultaneous iterative reconstruction technique (SIRT). The gray levels in DART reconstructions will vary depending on a number of factors such as imaging conditions and sample composition, therefore choosing them is an important step in the reconstruction process. The relative reconstruction quality can be determined by examining the projection difference, which is a measure of sum of the least-squares difference between the forward-projected reconstruction (line integrals through the reconstruction to form a projected image) and the acquired 2D images. Improving the projection error means that the final reconstruction is closer to the measured data. Therefore, by minimizing this, and maintaining realistic constraints on the reconstruction, the reconstruction resulting in the lowest projection error is likely the most accurate.

The projection error of the reconstruction was minimized by using only two gray levels, one for the polymer-rich domains, one for the fullerene-rich domains. The use of a third gray level couldn't be justified from what was known about the sample and some quick tests showed that the projection error was not improved much by the addition of a third gray level.

Since the intensity of the 2D images in annular dark-field scanning transmission electron microscope (ADF-STEM)images is dependent upon the composition, the reconstruction gray level will represent the local composition of the sample. Using simple algebraic relationships, the composition of each grey level can be determined. Unfortunately, composition calculations for the different regions weren't possible with this sample because the measured area was likely not large enough to have the same average composition of the sample, which is a requirement for such calculations. However, by combining the STEM/EELS and ET, it was

determined that the fullerene-rich domains were ∼80 % fullerene, the polymer-rich domains were likely 100% polymer because of the low miscibility of the endohedral fullerene.

GIXD

The local scattering intensity distribution near different features in the GIXD data for PTB7:$Lu_3N@C_{80}$-PCBEH films with different additives are shown in figures S2-S5, only the DIO additive films show any strong peaks in the distributions shown.

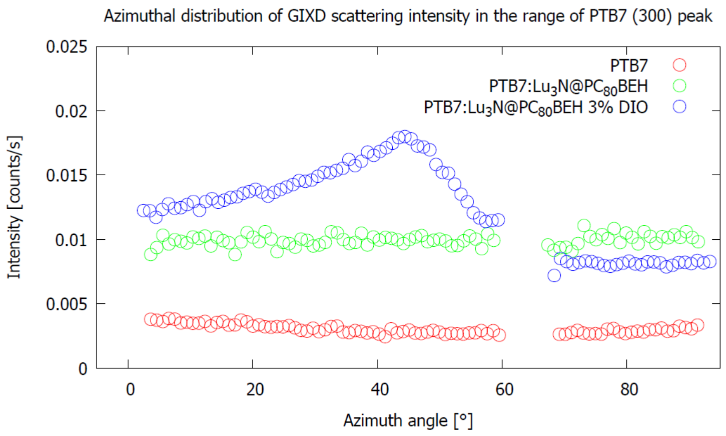

Figure 2: Azimuthal distribution of scattering intensity near the PTB7 (300) peak.

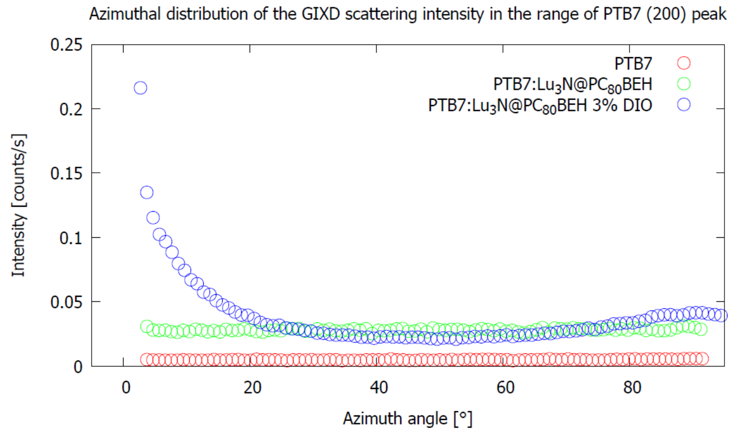

Figure 3: Azimuthal distribution of scattering intensity near the PTB7 (200) peak.

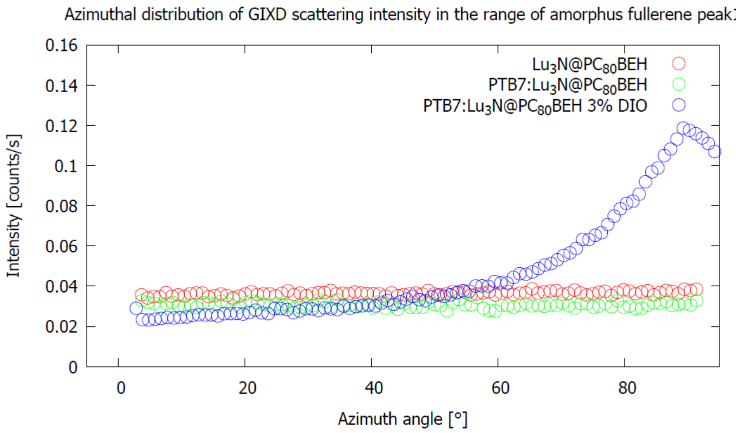

Figure 4: Azimuthal distribution of scattering intensity near the first amorphous fullerene peak

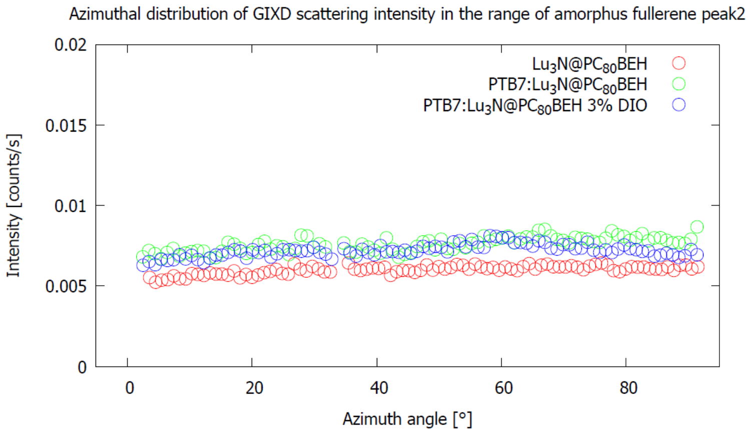

Figure 5: Azimuthal distribution of scattering intensity near the second amorphous fullerene peak.

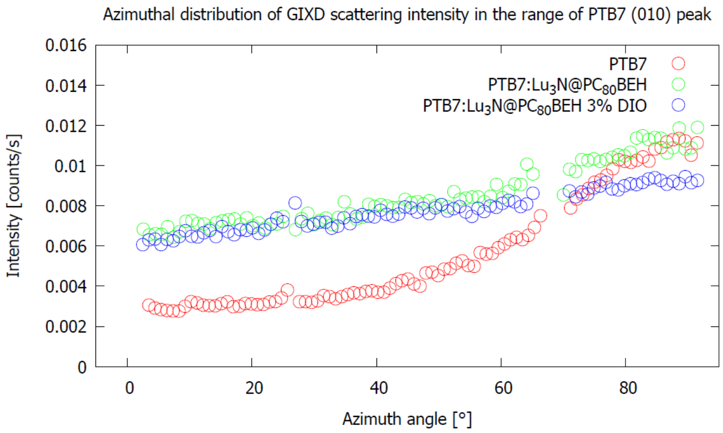

Figure 6: Azimuthal distribution of scattering intensity near the PTB7 (010) peak.

Fluorescnece Quenching

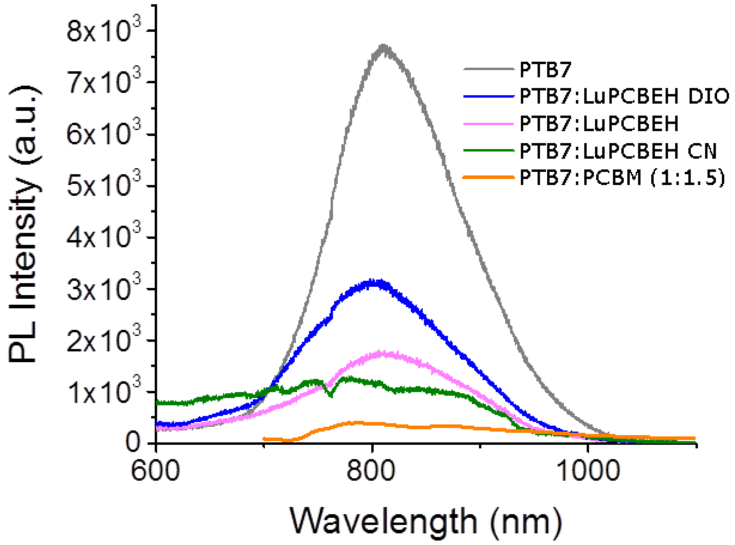

Figure 7: Photolumenescence data of PTB7/$Lu_3N@C_{80}$-PCBEH films processed under various conditions

This data largely verifies the large scale phase separation in DIO additive and no additive films with minimal fluorescence quenching, less for the DIO additive film. It also shows some improved mixing with the CN additive film as also seen in the images. The fluorescence quenching in $PC_{60}BM$ films is higher than any $Lu_3N@C_{80}$-PCBEH film. This result may indicate that the LUMO level of the $Lu_3N@C_{80}$-PCBEH is not sufficiently lower than the LUMO of the PTB7 to cause quantitative exciton separation at the donor/acceptor interface.
